# Supplementary material for: Closed for business: The mortality impact of business closures during the Covid-19 pandemic
Source: PLoS One. 2021 May 14;16(5):e0251373. doi: 10.1371/journal.pone.0251373 (PMC8121299; doi:10.1371/journal.pone.0251373)
Supplement: S1 Table — This table shows the definition of each variable used in the empirical analysis. (PDF) [file pone.0251373.s001.pdf]

S1 Table: Variable Definition

Variable	Definition
$ExcDeathRate_{m,t}$	Number of deaths in day t of 2020, minus 7-day average around t of 2015-2019, divided by municipality's residents;
$\Delta ExcDeathRate_{m,t,t-1}$	Daily change in $ExcDeathRate_{m,t}$
$gExcDeathRate_{m,t,t-1}$	Daily growth rate in $ExcDeathRate_{m,t}$;
$log(cExcDeathR_{m,t}+1)$	Cumulative $ExcDeathRate_{m,t}+1$, natural log;
$d11_t$	Dummy variable, taking a value of one as of ten days after first policy (03/21); zero otherwise;
$d25_t$	Dummy variable, taking a value of one from ten days after second policy (04/05); zero otherwise;
$Shutdown11_m$	Number of employees and business owners of sectors shutdown on March 11th divided by the total employees and business owners in a municipality m;
$Shutdown25_m$	Number of employees and business owners of sectors shutdown on March 25th divided by the total workforce in a municipality m;
$Food11_m$	Workforce in the food sector divided by the total labor force in a municipality m;
$Retail11_m$	Workforce in the retail sector divided by the total labor force in a municipality m;
$Personal11_m$	Workforce in the personal-services sector divided by the total labor force in a municipality m;
$log(Pop_m)$	Municipality m residents, natural logarithm;
$PopDens_m$	Residing population in municipality m per territory (squared km), natural logarithm;
$IntMob_m$	Internal work/study inflows in the municipality m, ratio of inflows and outflows from m;
$HighSchool_p$	High school graduates, fraction of working age population;
$IncIneq_m$	20-20 income fraction of working age population in a municipality m;
$Elderly_p$	Number of 65+ old people, fraction of population;
$Hospitaliz_p$	Number of beds available in all hospitals per residents in a province p, percentage points;
$RelShutdown11_n$	$Shutdown11_m$ of the largest exposed municipality m relative to the total workforce in m, with at least 16,500 inhabitants, in the province;
$AbsShutdown11_n$	$Shutdown11_m$ of the largest exposed municipality m in absolute terms (number of affected workforce), with at least 16,500 inhabitants, in the province;
$PopShutdown11_n$	$Shutdown11_m$ of the largest exposed municipality m in terms of number of residents, with at least 16,500 inhabitants, in the province;
$WinterTourists_p$	Number of tourists in all types of accommodations in a province p, over January-February 2007 (ISTAT);
$WeekArrival_m$	Time from the virus arrival date in a municipality m to March 21th, measured in number of weeks;
$DaysArrival_{m,t}$	Number of days elapsed from the virus arrival date in a municipality m;

This table shows the definition of each variable used in the empirical analysis of the paper *Closed for Business: the mortality impact of business closures during the Covid-19 pandemic (Bongaerts, Mazzola and Wagner, 2021).*
