# Supplementary material for: Closed for business: The mortality impact of business closures during the Covid-19 pandemic
Source: PLoS One. 2021 May 14;16(5):e0251373. doi: 10.1371/journal.pone.0251373 (PMC8121299; doi:10.1371/journal.pone.0251373)
Supplement: Attachment — Submitted filename: Priyankas review with track changes.docx [file pone.0251373.s002.docx]

Final Review

1) The authors should revise the language to improve readability.

I have attached few pages for which I checked for grammatical errors and have stated them in the form of track changes.

I would request that the authors advise the authors work with a writing coach or copyeditor to improve the flow and readability of the text.”

Few pages with track changes

In attempts to contain pandemics, policy makers trade off public health benefits against

economic costs. Yet, little is known about how containment policies should be designed,

or how they should be organized. Countries have in fact varied widely in their responses

to the outbreak of the novel coronavirus (SARS-CoV-2). Some governments have closed

almost their entire economy for a certain period of time, while many others have chosen

selective closures of varying breadth. In some countries, policies were predominantly

decided upon at the regional level, whereas in others at the national level. Containment

interventions are also not necessarily orchestrated by public authorities. For example,

the first lockdowns in Brazil were imposed by drug gangs in favelas in Rio de Janeiro.As

measures are selectively reversed and reintroduced, there has been a recent tendency for

more and more localized approaches, often going hand-in-hand with a decentralization of

decision-making. At the extreme end, laissez-faire approaches (such as the one followed by

Sweden) fully decentralize decisions, by relying on individual businesses and households

to curb the spread of the virus.

The empirical examination of containment policies is challenging, for several reasons

(Goodman-Bacon and Marcus, 2020). Containment measures are typically implemented

in response to a rapidly evolving pandemic. Therefore, there are no clear counterfactuals

to these policies – the spread of the virus would also have changed in the absence of such

policies. Containment decisions are also often clustered, making it difficult to isolate the

impact of a specific policy. Policies tend to be introduced around the time that the

general public becomes acutely aware of the dangers of a virus, and takes (self-imposed)

measures to reduce the risk of contracting the virus.

This paper exploits within-country variation in exposure to a nationwide containment

policy to study the health impact of business shutdowns during a pandemic. During the

2020 Covid-19 pandemic, Italy was the first European country to shut down parts of its

economy. Specifically, on March 11th the Government first shut down sectors comprising 17.6% of the economy (in terms of employment); on March 25th additional sectors comprising 32.5% were closed. Importantly, this “one-size-fits-all ”closure policy affected Italian municipalities differently due to heterogeneity in sectoral exposures. Our empirical strategy is to study differential changes in mortality patterns across municipalities, allowing to control for confounding factors taking place in Italy around the time of the policy.

The results suggest that business shutdowns are effective in saving human lives: municipalities with higher exposures to the first shutdown see a decline in mortality rates

relative to other municipalities. Based on our estimates, we undertake a counterfactual

analysis that shows that the first shutdown saved about 9,500 Italian lives over a period

of 24 days. Estimates of the value of a statistical life-year imply a large societal benefit

from business closures, exceeding nine billion Euros. Interestingly, we find that the second shutdown is not as effective (per unit of employment closed down) as the first one,

supporting the notion that the first shutdown already reduced the spread of the virus.

Our analysis also shows that business shutdowns have important mortality spillovers.

Mandated business closures may affect the spread of the virus outside a municipality

because of commuting (other forms of travel were fairly restricted during our sample

period). Consistent with this hypothesis, we find that shutdown exposure in business

centres affects mortality rates in neighboring municipalities. The beneficial effect is large,

and comparable in size to the impact of a municipalities’ own shutdown exposure. We

also find that greater shutdown exposure has a strong effect on parts of the population

that is very unlikely to work (e.g., the elderly). This points to significant contagion effects

that reach beyond the firms undertaking economic activities. The existence of different

forms of spillovers suggest a need of coordinating shutdown decisions at a central level.

Finally, our analysis points at rapidly declining benefits to scale from sectoral shutdowns. We compare the marginal effectiveness of shutdowns across municipalities that

differ with respect to the proportion of their economy that was affected by the first
shutdown. We find the marginal effectiveness in municipalities with the lowest sector

exposure to be about three times higher than the average marginal effectiveness across

all municipalities. Since the (marginal) effectiveness also varies across sectors (we find

per-unit shutdowns in some sectors to be more effective than in others), our evidence

speaks broadly in favour of targeted closures of a select number of sectors, rather than

more uniform shutdowns.

In the wake of the Covid-19 crisis a significant theoretical literature is emerging that

examines optimal policies during a pandemic. This literature emphasizes production

externalities as a rationale for public policies. Production externalities arise when the

provision of goods and services result in the spread of the virus to individuals not directly

involved in the business activities. As firms (and their workers) will not internalize the

social cost of such contagion, they will make inefficient containment decisions, providing a need for government-imposed shutdowns. Our paper is the first, to the best of

our knowledge, to document empirical evidence consistent with such production externalities. Eichenbaum et al. (2020) study optimal taxation of business activities (which

can be interpreted as shutdown intensity) in an environment where the severity of the

production externalities vary with the spread of the virus. Our finding of lower policy

effectiveness once the pandemic is more under control is consistent with the theoretical

premises of their model. Krueger et al. (2020) show that production externalities are

mitigated by individuals shifting activities to environments that pose less contagion risk.

Our estimates – which are net of such mitigating behavior – suggest that production

externalities remain significant. Calibrating an SIR-model to the US economy, Bethune

and Korinek (2020) show that the social cost of infections exceeds the private cost by

factor two, consistent with our results of large spillovers on individuals that are unlikely

to work. Beck and Wagner (2020) analyze virus contagion across jurisdictions

(countries, in their model) and show that inter-jurisdictional externalities creates a need

for coordination of containment policies. Our findings of strong geographical spillovers provide empirical evidence for the existence of such externalities.

The various government approaches taken in response to pandemics are spurring a

rapidly evolving literature that tries to understand their benefits, as well as their costs.

On the benefits side, several papers have examined the impact on mobility, infections

and/or ultimately on mortality. Using predominantly time-series and/or cross-country

variation, these studies have generally concluded that these government interventions

are effective. See for example Adda (2016) for France; Hartl et al. (2020) for Germany;

Qiu et al. (2020) for China; Correia et al. (2020), Chernozhukov et al. (2020), Sauvagnat

et al. (2020) and Dave et al. (2020) for the US; Cho (2020) and Born et al. (2020) for a

counterfactual analysis for Sweden; Ullah and Ajala (2020) and Dergiades et al. (2020)

for a cross-country study.For a collection of working papers, see Baldwin and Weder di

Mauro (2020).Our study, using within-country variation in exposure to national business

shutdowns in Italy, confirms and quantifies the effectiveness of containment measures.

In addition, we show that business shutdowns have first-order spillover effects as well as

declining marginal returns. These results provide valuable information for policymakers

in their ongoing challenge of devising and adapting containment policies.

Methodology and Data

On March 11th 2020 the Italian Prime Minister mandated to shut down all food, retail and personal-services activities. Businesses like supermarkets, small grocery shops,

pharmacies, and newsstand kiosks were allowed to remain open. The national decree

also introduced restrictions on personal mobility. Two weeks later, on March 25th, the

list of sectors included in the shutdown policy was enlarged.

For reasons of identification, our study primarily focuses on the first policy. We

study the impact of this shutdown on mortality rates using a difference-in-differences

(hereafter diff-in-diff) approach with continuous treatment. Given the lag between a

5

virus infection and a (possible) subsequent death, the “treatment” date does not coincide

with the day the policy was enacted. Actually, it is impossible for the policy to have any

effect on mortality already from its implementation date. Estimates in the literature

suggest a median of five days between the exposure to the virus and the occurrence of

first symptoms (” the incubation” period, see Lauer et al., 2020) and about eight days

between first symptoms and death at that time. See for example of Health (2020). Thus,

the median time to death across individuals is about 13 days. Due to dispersion, any

effective policy will arguably produce its effects 2 or 3 days before this date; we therefore take the “treatment date” to be day 10.

Our empirical model takes the following form

ym,t = βym,t−1 + d11t + φ(d11t × Shutdown11m)+

+ d25t + ψ(d25m × Shutdown25m) + θ(d11t × Controlsm) + F Em,tsa,t + εm,t (1)

where ym,t is the (Covid-related) mortality rate in municipality m on day t. We include

the lagged value, ym,t−1, as a determinant since epidemiological models (such as the SIR

model in e.g., Kermack and McKendrick, 1927; Allen, 2017) show that new infections

condition highly on the prevalent share of infected people in the population. We include

two dummies, d11t and d25t

, to indicate the treatment date for the first and second shutdown, both taking value of one as of the tenth day after their respective announcements.

The variables Shutdown11m and Shutdown25m measure the exposure of a municipality

to sectors that were (incrementally) shut down at the first and second shutdown, respectively. Our variable of interest is the interaction coefficient φ, which captures whether

municipalities with a higher shutdown exposure experience lower daily mortality rates

as a consequence of the policy. If the policy shutdown is effective at reducing the spread

of the virus and thus, ultimately, reduces mortality, then the prediction is that the coefficient φ will enter with a negative sign. We saturate the model with municipality and

2) Authors have chosen a very relevant topic in the current scenario where they are discussing effect of closure of major business during the current pandemic.
3) They have followed good Data Analyses and methodology for data interpretation.
4) Their funding’s can be great help to policy makers all over the world.
