# Supplementary material for: Closed for business: The mortality impact of business closures during the Covid-19 pandemic
Source: PLoS One. 2021 May 14;16(5):e0251373. doi: 10.1371/journal.pone.0251373 (PMC8121299; doi:10.1371/journal.pone.0251373)
Supplement: Attachment — Submitted filename: Response to Reviewers_Referee2.docx [file pone.0251373.s003.docx]

­Dear referee,

Thank you so much for taking the effort to carefully read our paper and provide us with thoughtful and constructive comments and suggestions. We are excited to have had the opportunity to revise our paper for potential publication in PLOS ONE. Over the last weeks, we have incorporated your comments into an improved version of the paper. Below, we provide a detailed response to your comments. For our responses, we will repeat your comments (in italics) and carefully indicate how we addressed each of your comments and where in the paper these changes can be found (in regular font). All references to pages and sections are based on the new, revised version of the paper, unless indicated otherwise.

Best regards,

The authors

­­

# Response to comments of Referee 2

*General Evaluation:
I found this to be an interesting, timely paper with interesting policy implications. The methods are generally well-defended.*

Thank you.

*However, there are some points where I believe additional explanation or slight changes in method would be warranted, discussed in the section below.
Recommendations:
I have divided by recommendations into “major” recommendations and “minor” recommendations, based on my view of their importance.*

Thank you. We address all of them below.

*Major Recommendations*

1. *Page 7 – The paper says that shutdown exposure is constructed by “dividing the number of employees and employers in sectors shut down… by the total number of employees and employers in the municipality.” It’s unclear why employees are being added to employers, as this seems to be mixing together two very different units (people and firms). If there are differences in typical firm sizes between municipalities and if shutdowns have a disparate impact on firms of a specific size, this could lead to some strange behavior in this variable, as the number of firms would be heavily weighted in importance in municipalities with very small firms and receive less weight in municipalities with fewer, but larger firms. I recommend constructing the Shutdown variables on the basis of employees alone (or at least using this alternative construction as a robustness check).*

Thank you for this comment. We realize our exposition was not clear. Both employees and employers refer to the actual people working in the business (and we are thus not mixing people with firms). Employers, in particular, according to the ISTAT definition, refers to the number of business owners (including their partners/relatives) that are present everyday at work. As both employees and employers are in principle susceptible to contracting the virus, hence we include them both.

We have modified our writing and now refer to “business owners” to avoid the confusion between firms and people.

It would indeed be interesting to examine if and how our results change if exclude the business owners (as, for example, business owners may be more active and hence more likely to contract the virus than an ordinary worker, resulting in different coefficients) but unfortunately the ISTAT data does not separate out both types (at the municipality-industry level).

2. *Page 10 – The paper defines the business centre as “the municipality with the highest share of closed sectors among the province’s larger municipalities (with at least 16,500 inhabitants).” It’s unclear why a high percentage of closed sectors would imply that a municipality is a business centre. Why not the municipality with the greatest number of inhabitants? Or the greatest number of employees (rather than share of employees) in the shutdown sectors? Explaining the reasoning behind this construction would be helpful.*

Thank you for this comment. Confusion may arise due to our usage of the term “business centre” in the previous version of the paper, which have been misleading. We intend to study whether there are any spillovers from the closure policies. Clearly, the potential for spillovers is the highest if a municipality is large, and if it has many closed down businesses (a combination of the size and exposure criteria). The town/city that is commonly referred to as “the business centre of the province” does not need to satisfy necessarily both criteria (e.g., the “real” business centre may be a municipality with many businesses but few of them being affected by the policy).

To make sure that our results would be robust to different definitions of business centers, we now also investigate the robustness of our spillover results. In the previous version of the paper, we looked for spillovers from municipalities with the highest relative exposure to the policy within their respective provinces, while satisfying a minimum size criterion. We now also look at spillovers from the town/city with (i) the highest absolute number of workers affected by the policy, and (ii) the highest number of residents in a province. We include these results in table 5 (columns 4 and 5). If anything, we find even stronger effects with these alternative definitions. We have adjusted the writing in the paper to reflect these different definitions on page 6.

*Minor Recommendations*

1. *Page 2 – first paragraph - Was Sweden’s approach really “laissez-faire”? My understanding is that they did make some attempt to isolate the most at-risk populations and to restrict the sizes of gatherings.*

Thank you for this comment. We understand that these “restrictions on gathering sizes” were actually recommendations, not legal requirements. After our initial submission of the manuscript, Sweden introduced some more measures, like face masks (still recommended, not required). See also <https://doi.org/10.1016/S0140-6736(20)32750-1>

We agree with the referee that, given the policy in Sweden as of now, “laissez-faire approaches” is too strong. We have hence rephrased into “laissez-faire-like approaches” to soften the statement.

2. *Page 6 – the phrase “Due to dispersion” is unclear to me. I’m guessing it just means that there will be variation around the median, but, in the context of an epidemic, I wonder if “dispersion” has an epidemiological meaning that I’m not familiar with.*

It indeed means that there is variation around the mean. We replaced “dispersion” by “variation around this mean” to avoid any confusion.

3. *Page 6 – Equation 1 – I’m not sure why there aren’t parameters put on the dummy variables for d11t and d25t. It makes no difference to the substance.*

Thanks for pointing this out. We put coefficients in front of *d11_t_*  and *d25t*.

4. *On page 11-12 – The paper describes the construction of Figure 1 by discussing dividing municipalities into cohorts based on their own stage in the pandemic. Then, “Within each of these cohorts, we calculate the average excess mortality rates for municipalities above and below the median shutdown exposure, and then average across cohorts.” This process seems like the only effect it would have is overweighting the small cohorts, it’s not obvious how this corrects for the stage of the pandemic. More explanation would be helpful.*

We realize that our exposition was not precise, causing this confusion. Our approach indeed corrects for the stage of the pandemic. Let us illustrate with an example.

Suppose mortalities in high- and low-exposure municipalities in early and late stages of the pandemic are as follows

|  | early | late |
| --- | --- | --- |
| High exposure | 1 | 10 |
| Low exposure | 2 | 11 |

We can see that high-exposure munis have lower mortality rates in each group. Suppose now that 10% of the high exposure munis are in the early stage (thus 90% in the late stage), whereas for low-exposure municipalities it’s 50/50.

A simple average of mortalities across munis would thus lead us to (incorrectly) conclude that mortality is higher in high-exposure munis (10%*1+90%*10-50%*2+50%*11=2.6). We instead calculate first differences for each pandemic stage (-1 for both early and late) and then average (still -1), showing lower mortality in high-exposure areas.

This approach is similar to portfolio-sorting in asset pricing, where the characteristics of portfolios are compared once they have been conditioned on a variable (here: pandemic stage). Conceptually, this is similar to a multivariate regression where pandemic-stage is a control variable. In our regressions, we also do control for the stage of the pandemic. Because such effects are likely to be non-linear in measures of the stage of the pandemic, we do this using granular pandemic stage fixed effects.

We now describe our approach more carefully on page 7.

5. *On page 20, near the end of the first paragraph – the “Shutdown25” has inconsistent subscript use between the 2 and 5. I think it should be “Shutdown25” with just an “m” in the subscript.*

Thanks for pointing this out. That is indeed correct. We corrected the typo.
